# Patient Handover in Emergency Trauma Situations

**DOI:** 10.7759/cureus.9544

**Published:** 2020-08-04

**Authors:** Munawar Peer, Noel B O'Regan, Bradley Evans, Amanda Fowler, Adam Dubrowski

**Affiliations:** 1 Medicine, Memorial University of Newfoundland, St. John's, CAN; 2 Anesthesiology, Memorial University of Newfoundland, St. John's, CAN; 3 Surgery, Memorial University of Newfoundland, St. John's, CAN; 4 Health Sciences, Ontario Tech University, Oshawa, CAN

**Keywords:** trauma, resident simulation, general trauma surgery, emergency

## Abstract

Miscommunication during patient handover can be a major cause of preventable medical errors. Emergency traumas are situations where high stress and cognitive load make communication more difficult. Simulation allows for junior learners to practice emergency scenarios in a low-risk setting. This technical report outlines a simulation involving patient handover in emergency trauma scenarios. The intended group of learners are first-year surgery and emergency medicine residents. The scenarios were developed based on the learning objectives of communication, collaboration, and information transfer. Using a high-fidelity simulation mimicking a tertiary care facility, the skills performed in these scenarios can be applied to everyday practice.

## Introduction

According to a report by the Joint Commission, an estimated 80% of serious medical errors are related to miscommunication during patient handover [1]. In a survey conducted in a US hospital, 59% of internal medicine and general surgery physicians reported that they had witnessed harm to one or more patients due to poor patient handover [2]. Patient handover is the transfer in responsibility in the care of a patient from one person or group to another [3]. It can occur within a single hospital setting during a shift change or when a patient is transferred between units. It can also occur when a patient is transferred between medical facilities such as when a patient is transferred to a Level I trauma center. The skill that this technical report intends to teach is patient handover during an emergency trauma situation between emergency medicine and surgical residents. Patient handover involves skills such as communication, collaboration, and information gathering.

Individuals of different backgrounds and knowledge bases are involved in the treatment of patients. These individuals must collaborate with one another using their expertise in order to reduce any knowledge gaps. This makes clear and efficient communication vital, as necessary information must be transmitted to the person assuming care. A medical professional that assumes care also has to rely on working memory, as they have the responsibility to gather and retain this information [4]. Tools such as cognitive aids could potentially assist in the retention of knowledge and help streamline the process. Practicing these skills through simulation can allow for junior learners to identify the best strategies to use in real scenarios. These skills are ultimately important, as gaps in communication can contribute to a lack of continuity in care, which can result in poor patient outcomes [5].

The intended groups of learners are first-year surgery and emergency medicine residents in a tertiary care facility. These groups of learners often face situations where they will receive patients with little to no knowledge about their condition. It is their responsibility to collect any vital information and transfer this information to the next person that will assume the care of the patient.

This technical report outlines a novel simulation that involves both patient handover and an emergency trauma situation. Descriptions of simulation exercises involving emergency trauma situations already exist [6], but they do not incorporate the patient handover component. Also, there is literature related to protocols for creating efficient patient handover in trauma situations [7]. This includes the development of trauma handover checklists developed by medical professionals, but these lists have not yet been integrated into mainstream use. A number of patient handover simulations have also been conducted on medical students, examining aspects like working memory, cognitive load, collection of information, and communication protocols [4]. This report can build upon the existing literature by examining communication, collaboration, and information gathering through the lens of an emergency. What makes trauma scenarios unique is that they occur in a fast-paced high-stress environment where time is limited. This study also looks into the effectiveness of cognitive aids to make this process more efficient.

Learning objectives for this simulation are related to developing skillsets involving communication, collaboration, and information transfer and gathering in a scenario related to emergency trauma.

## Technical report

Case

Run 1: A 23-year-old male involved in an all-terrain vehicle (ATV) accident just arriving in the trauma bay. The paramedic will be briefed to give handover to the trauma leader (Learner #1). Primarily abdominal injury. Learner #1 will hand over to Learner #2.

Run 2: A 30-year-old male fell from a ladder onto a fence. The paramedic will be briefed to give handover to the trauma leader (Learner #1). Primarily abdominal injury. Distractor inserted (Jehovah's Witness Card) during the handover from Learner #1 to Learner #2.

Run 3: A 30-year-old male ATV accident without a helmet. The paramedic will be briefed to give handover to the trauma leader (Learner #1). Primarily head injury. Distractor will be patient coning during the handover from Learner #1 to Learner #2.

In this technical report, educational context, inputs, processes, and expected products are elements that will support this simulation-based scenario. These elements are organized into a modified context, input, process, and product (CIPP) program development and evaluation model [8].

Context

The appropriate setting for this simulation is a hospital or a university-based simulation facility. This type of facility most accurately models a tertiary care facility in which residents would receive their training and face an emergency trauma situation. This is a hybrid simulation taking into consideration both technical and communication skills [9].

Input

Equipment

Tables 1-3 outline the equipment that will be available in the room during the duration of each simulation run.

**Table 1:** Simulation lab set-up CT: computed tomography; MRI; magnetic resonance imaging

Patient simulator/task trainer:	3G patient simulator
Environment/setting/location of scenario:	Trauma bay
Specialty supplies/medications/infusions required:	Normal saline, ringers lactate, blood, tranexamic acid, Ancef, mannitol, hypertonic saline, norepinephrine infusion, epinephrine, laryngoscope and endotracheal tube, bed that can be flexed to 30 degrees, morphine, fentanyl
Supporting files (labs/X-rays/CT/MRI reports):	Chest X-ray, pelvic x-ray, complete blood count, electrolytes, blood urea nitrogen, creatinine, liver function tests, coagulation tests, type and screen, focused assessment with sonography for trauma

**Table 2:** Monitors required a=available; x=on patient at start of scenario BP: blood pressure; ECG: electrocardiogram

Monitors Required			
x	Non-invasive BP	a	Capnography
x	ECG rhythm (sinus tachycardia)	a	Urinary catheter
x	Heart rate	a	Temperature
x	Oxygen saturation	a	Arterial line

**Table 3:** Time duration per scenario

Stage	Time (mins)
Setup	15
Simulation	10
Debriefing	20

Personnel

Table 4 outlines the personnel that will be available to assist the learner in the room upon commencement of the simulation.

**Table 4:** Personnel

Personnel
Paramedic
Will be at bedside when trainee enters room.
Nurse 1
Responds to prompts by trainee. Can suggest leads and oxygen if not prompted within first 60 seconds.
Nurse 2
Responds to prompts by trainee.
Patient (Run 1)
Arms around abdomen in bed. Screaming in pain until treated. Responds to questions about past medical history as above.
Patient (Run 2)
Arms around abdomen in bed. Screaming in pain until treated. Responds to questions about past medical history as above.
Patient (Run 3)
Unconscious. No eye opening. Incomprehensible sounds. Withdraws from pain only.

Process

Pre-Briefing

Learners will be provided with an orientation of the simulation lab and the available equipment. A general overview of the simulation will be explained. The formative nature of this simulation and how it will only be used for teaching purposes without a pass/fail system will be explained.

The Simulation

**Figure 1:**
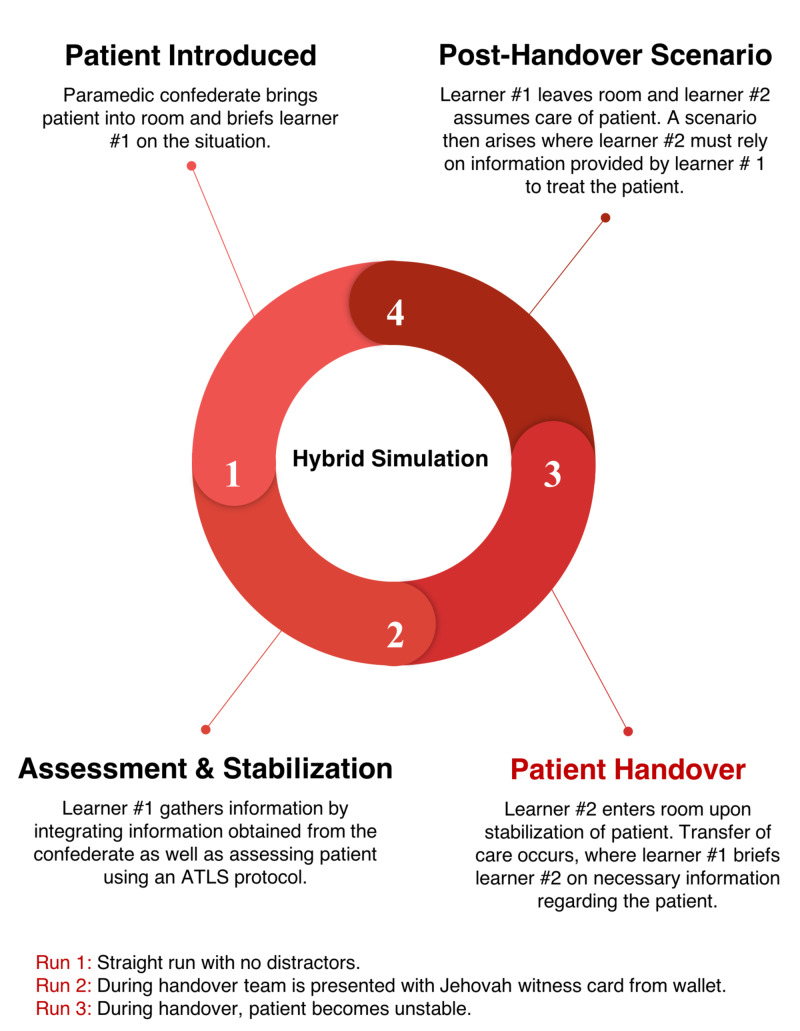
Process Overview

**Figure 2:**
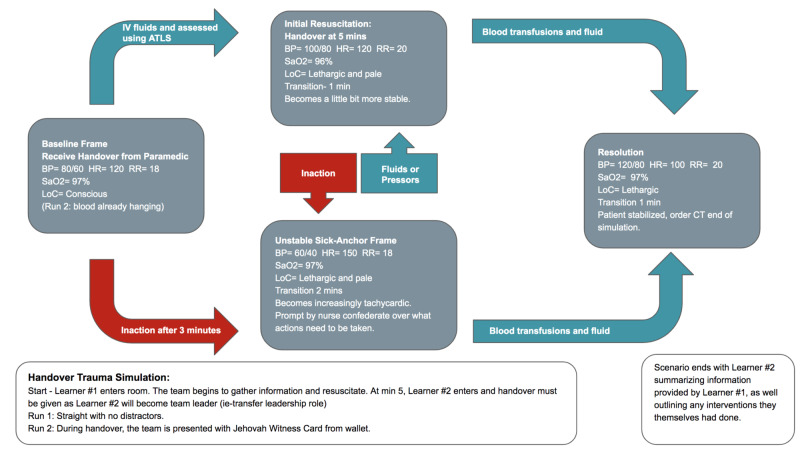
Storyboard for Runs 1 & 2

**Figure 3:**
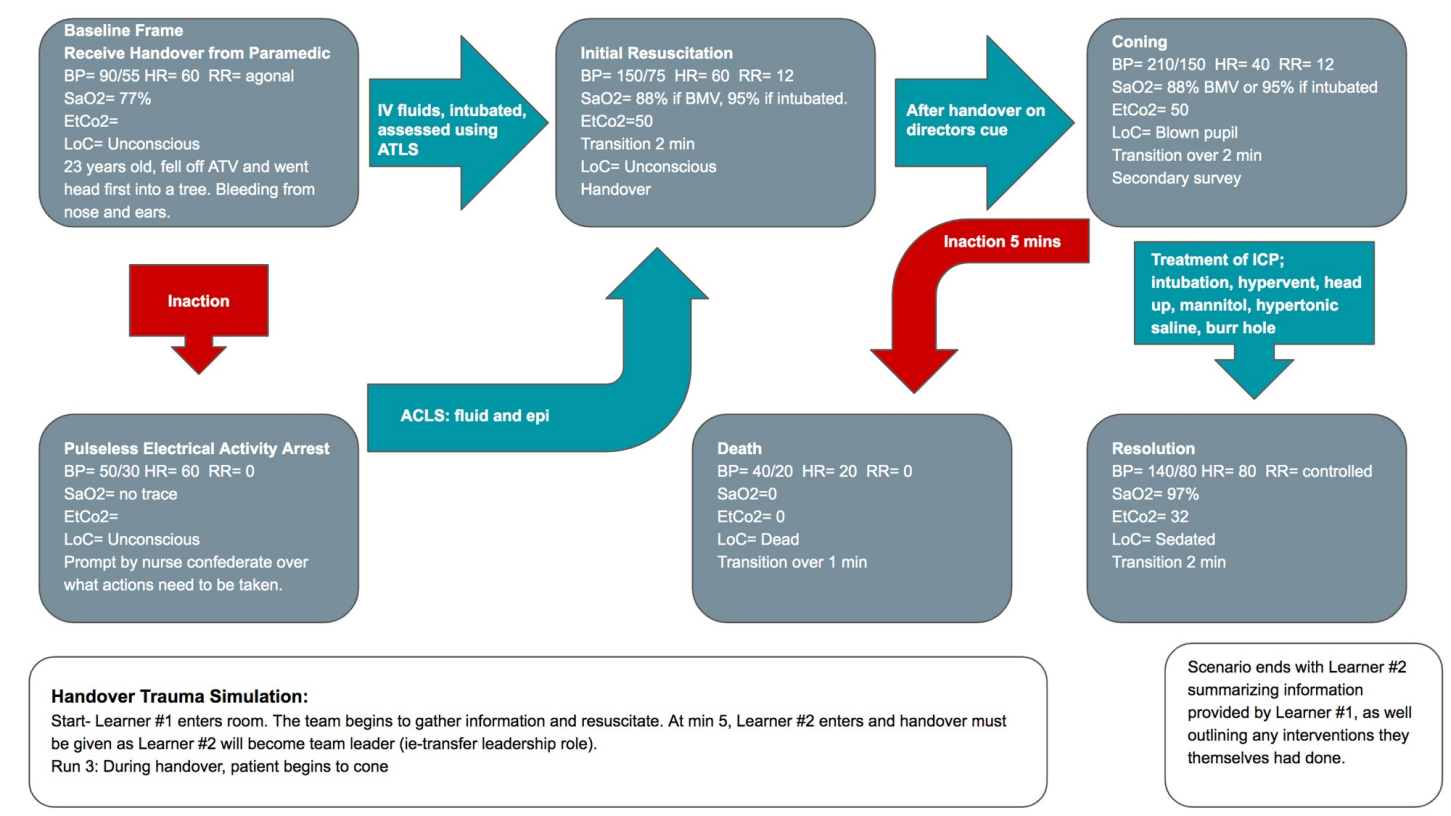
Storyboard for Run 3

The scenario begins with a standardized patient brought into the room with a paramedic confederate briefing Learner #1 on the situation. One nurse confederate will also be at hand in the room to provide any assistance the learner may require. The first stage involves information gathering, with Learner #1 integrating the information obtained from the paramedic confederate, as well as assessing the patient, using the Advanced Trauma Life Support (ATLS) protocol. A handover checklist developed based on the acronym SBAR (Situation, Background, Assessment, Recommendation) is present in the room as a cognitive aid, but its use is optional. Upon the collection of necessary information and stabilization of the patient, Learner #2 enters the room. Care of the patient is transferred from Learner #1 to Learner #2. During this transfer, Learner #1 communicates any necessary information regarding the patient to Learner #2. This also gives Learner #2 the opportunity to ask any relevant questions before assuming the care of the patient. After Learner #2 assumes care, a second scenario arises and Learner #2 will need to rely on the information given to them by Learner #1 to treat the patient. Upon completion of the simulation run, the learners will be debriefed. The learners will each complete three different scenarios, with expected improvement after each run. The scenarios of these runs are detailed in Figures 1-3 and Tables 5-7.

**Table 5:** Patient History Run 1 HPI: history of the present illness; ATV: all-terrain vehicle; LOC: loss of consciousness; PMHx: past medical history

Run 1	
HPI	“23-year-old male ATV accident approximately 30 mins ago. Witnessed by a friend riding on a separate ATV. No LOC at the scene, was wearing a helmet. Lost control and hit a large boulder at 30-40km/hr. Complaining of abdominal pain. Last blood pressure on route 100/70 mmHg and heart rate 115 beats per minute (BPM). C-collar in place. 500cc of normal saline given by paramedic.”
PMHx	Appendectomy
Social Hx	Lives with parents. Smokes half a pack of cigarettes per day, social ethanol use. No other drugs.
Family Hx	Nil significant
Medications	None
Allergies	None

**Table 6:** Patient History Run 2 HPI: history of the present illness; LOC: loss of consciousness; PMHx: past medical history

Run 2	
HPI	“30 year male fell from ladder onto fence while at work 30 mins ago. Witnessed by a co-worker. No LOC at scene. Impact primarily to abdomen. Complaining of abdominal pain. Last blood pressure on route 100/70 mmHg and heart rate 115 BPM. C-collar in place. 500cc of normal saline given by paramedic.”
PMHx	Von Willebrand Disease
Social Hx	Roofer. Nonsmoker. No ethanol use. No other drugs.
Family Hx	Nil significant
Medications	None
Allergies	Penicillin

**Table 7:** Patient History Run 3 HPI: history of the present illness; PMHx: past medical history; BPM: beats per minute

Run 3	
HPI	“23 year male ATV accident approximately 15 mins ago. Witnessed by a friend riding on a separate ATV. Not wearing a helmet, hit a tree at 30-40km/hr. Unconscious after incident. Oxygen saturation 77% - progressively worsening during transport. Last blood pressure on route 100/70 mmHg and heart rate 70 BPM. C-collar in place. 500cc of normal saline given by paramedic.”
PMHx	None
Social Hx	Fisherman. Smokes one pack of cigarettes per day, social ethanol use. No other drugs.
Family Hx	Nil significant
Medications	None
Allergies	None

Debriefing

Feedback will be based upon the debriefing with good judgment principles that involve self-reflection [10]. This will be heavily centered around the learning objectives of communication, collaboration, and information gathering. Learners will be asked to provide feedback on and reflect upon the simulation run. This could include aspects they felt they did well in and what they can improve upon. Furthermore, this provides an opportunity for the learners to suggest ways the simulation could be improved. An opportunity would then be given to confederates involved with the simulation to provide feedback regarding the simulation. Finally, two faculty members that observed the simulation would then provide feedback centered around the learning objectives. A brief discussion surrounding the importance of patient handover will then be discussed.

Products/outcomes

Learning Objective 1: Communication

Handover between Learner #1 and Learner #2 will be assessed based on adherence to principles of SBAR (see Appendix A) and the prehospital trauma handover checklist developed by Harmsen et al. [7]. Practices such as finding a quiet corner of the room to conduct the handover and using closed-loop communication will also be considered.

Learning Objective 2: Collaboration

Collaboration will be assessed using the Communication and Teamwork Skills (CATS) assessment instrument (see Appendix B). The checklist will include aspects like:

- How well Learners #1 and #2 collaborated during transfer (i.e., Learner #2 asking questions to clear doubts or Learner #1 giving recommendations).

- How well learners function within a team setting with nursing confederates.

Learning Objective 3: Information Gathering

Information gathering will be assessed by two faculty members observing the simulation and will take into consideration:

- How well Learner #1 examined the patient using an ATLS protocol

- How well Learner #1 integrates information relayed by the confederate into their care

- How well Learner #2 integrates knowledge attained from Learner #1 into their care

## Discussion

As junior learners inevitably face emergency trauma situations during their residency training, having the opportunity to practice in a controlled environment can ensure that they gain proficiency. In particular, emergency trauma handover poses a unique challenge due to the limited time frame, stress, and cognitive load associated with these events.

Learners are not only responsible for diagnosing and stabilizing a patient in a timely manner, but they must also be able to retain pertinent information and communicate this knowledge. As a result of these challenges, vital patient information is often omitted during the process of handover. Knowledge gaps during handover is a risk to patient safety and can result in poor health outcomes. However, with repeated practice with simulation scenarios, junior learners can better prepare themselves for real-life situations by utilizing skills they have attained.

This simulation teaches these skills based upon the three learning objectives and is supplemented by feedback from experienced faculty members. Furthermore, this simulation closely mimics real-life situations such that these skills are practical. By introducing aspects like distractors during handover that further increase the difficulty of handover, junior learners will be more prepared for random events that can complicate cases in real-life emergency departments.

## Conclusions

This technical report describes the design of a simulation scenario on patient handover during emergency trauma situations. Simulation provides an opportunity for junior learners to practice key skills related to handover in a controlled setting. These skills are based on the learning objectives of communication, collaboration, and information gathering. As the simulation mimics real-life scenarios, the skills attained can be practiced during encounters in the emergency department.

## Figures and Tables

**Figure 4:**
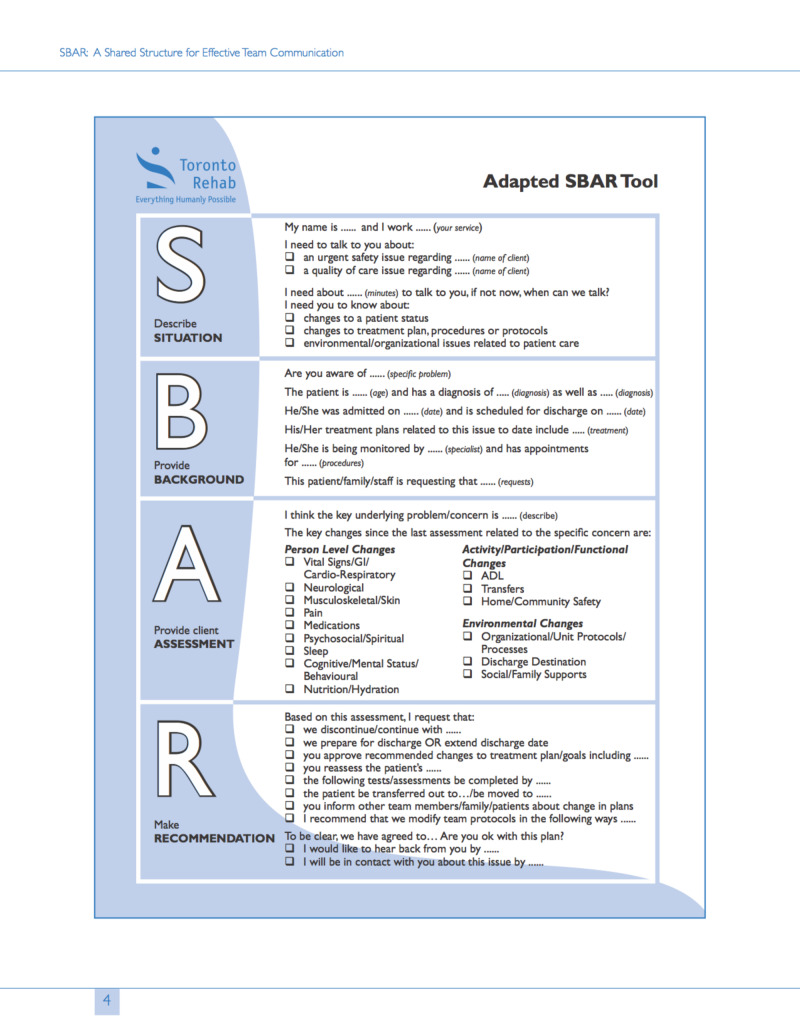
SBAR Toolkit Document Appendix A [11] Image reproduction approved with permission by author Angie Andreoli

**Figure 5:**
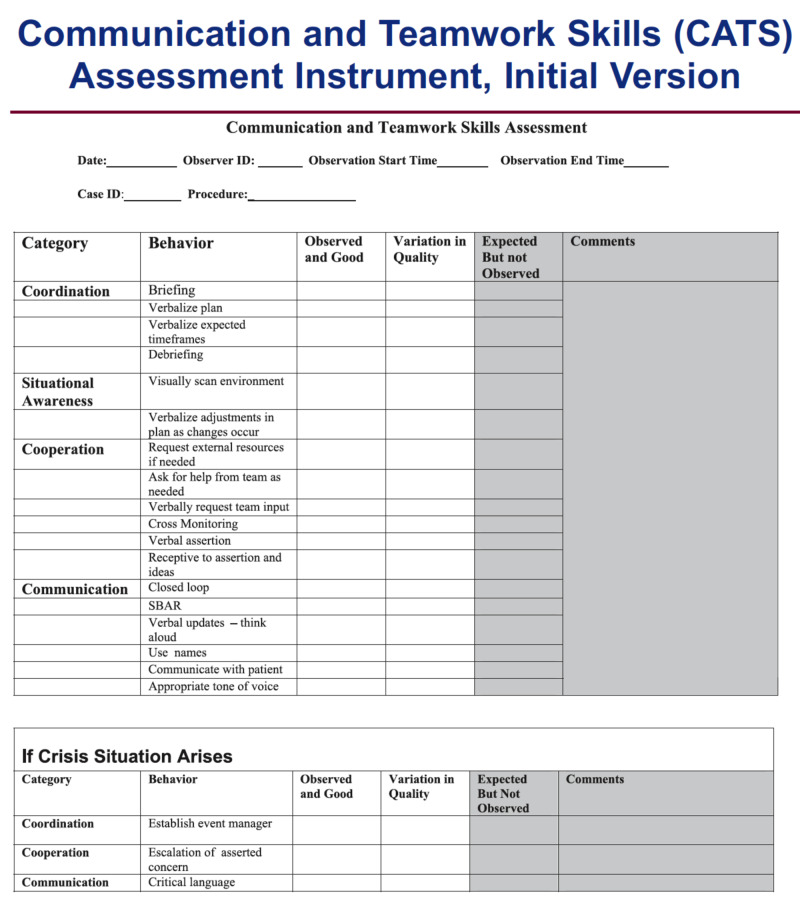
Communication and Teamwork Skills Assessment Instrument Appendix B [12] Image reproduction approved with permission by author Roxane Gardner.

## Appendices

Adapted SBAR tool 

Reproduction
